# Apoptosis as anticancer mechanism: function and dysfunction of its modulators and targeted therapeutic strategies

**DOI:** 10.18632/aging.100934

**Published:** 2016-03-27

**Authors:** Giuseppa Pistritto, Daniela Trisciuoglio, Claudia Ceci, Alessia Garufi, Gabriella D'Orazi

**Affiliations:** ^1^ Department of Systems Medicine, University “Tor Vergata”, 00133 Rome, Italy; ^2^ Department of Research, Advanced Diagnostics, and Technological Innovation, Regina Elena National Cancer Institute, 00158 Rome, Italy; ^3^ Department of Medical Oral and Biotechnological Sciences, Tumor Biology Unit, University “G. d'Annunzio”, 66013 Chieti, Italy

**Keywords:** apoptosis, defective apoptotic pathways, cancer, small molecules, miRNAs, p53

## Abstract

Apoptosis is a form of programmed cell death that results in the orderly and efficient removal of damaged cells, such as those resulting from DNA damage or during development. Apoptosis can be triggered by signals from within the cell, such as genotoxic stress, or by extrinsic signals, such as the binding of ligands to cell surface death receptors. Deregulation in apoptotic cell death machinery is an hallmark of cancer. Apoptosis alteration is responsible not only for tumor development and progression but also for tumor resistance to therapies. Most anticancer drugs currently used in clinical oncology exploit the intact apoptotic signaling pathways to trigger cancer cell death. Thus, defects in the death pathways may result in drug resistance so limiting the efficacy of therapies. Therefore, a better understanding of the apoptotic cell death signaling pathways may improve the efficacy of cancer therapy and bypass resistance. This review will highlight the role of the fundamental regulators of apoptosis and how their deregulation, including activation of anti-apoptotic factors (i.e., Bcl-2, Bcl-xL, etc) or inactivation of pro-apoptotic factors (i.e., p53 pathway) ends up in cancer cell resistance to therapies. In addition, therapeutic strategies aimed at modulating apoptotic activity are briefly discussed.

## INTRODUCTION

Apoptosis, the programmed cell death, is finely regulated at gene level resulting in the orderly and efficient removal of damaged cells such as those occurring following DNA damage or during development [1]. The machinery of apoptosis is complex and involves many signaling pathways. Apoptosis can be triggered in a cell through either the caspase-mediated extrinsic or intrinsic pathways. Both pathways converge to activate the effector apoptotic caspases resulting ultimately in morphological and biochemical cellular alterations, characteristics of apoptosis [2]. Usually, the balance between the pro-apoptotic and anti-apoptotic protein regulators is a critical key point to determine if a cell undergoes apoptosis. The induction of apoptosis as result of DNA damage in precancerous lesions can remove potentially harmful cells, thereby blocking tumor growth. Deregulation of this death process is associated with unchecked cell proliferation, development and progression of cancer and cancer resistance to drug therapies [3,4]. For that reason, deregulation of apoptosis is considered one of the hallmarks of cancer [5]. Therapeutic strategies targeting molecules involved in apoptotic resistance therefore represent a valid approach to be pursued in order to restore cancer cells sensitivity to apoptosis and overcome the ineffectiveness of the treatments [6,7]. This article focuses on the mechanisms of apoptosis, how defects along the apoptotic pathway contribute to cancer development and drug resistance and, briefly, how apoptosis can be used as a vehicle of targeted treatment in cancer.

### Morphological and biochemical changes in apoptosis

From the morphological point of view apoptotic cells show a characteristic cytoplasmic cell shrinkage, budding of plasma membrane, membrane exposure of phosphatidylserine (PS) on extracellular side, chromatin condensation and DNA fragmentation [8,9]. The plasma membrane is intact throughout the total process. The expression of PS in the outer layers of the cell membrane allows early recognition of dead cells by macrophages, resulting in phagocytosis without the release of proinflammatory cellular components [10]. At the later stage of apoptosis some of the morphological features include membrane blebbing, ultrastructural modification of cytoplasmic organelles and loss of membrane integrity [11]. Usually phagocytic cells engulf apoptotic cells before apoptotic bodies occur [12]. Apoptosis is primarily executed by a family of proteases known as the caspases (cysteinyl, aspartate-specific proteases) [13]. Caspases are central to the mechanism of apoptosis as they are both the initiators (caspase-2, -8, -9 and -10, primarily responsible for the beginning of the apoptotic pathway) and the executors (caspase-3, -6 and -7, responsible for the definite cleavage of cellular components) of cell death [14]. After being produced as inactive proteins (zymogens or pro-caspases), the initiator caspases auto-activate through auto-proteolysis, a process that is facilitated by their interaction with specific adapter molecules [15]. Once activated, the initiator caspases cleave off the executors caspases that perform critical cleavage of specific cellular substrates resulting in the final apoptotic cell death [16]. This caspases activity is responsible of the apoptotic hallmarks, such as chromatin condensation, plasma membrane asymmetry and cellular blebbing. The extensive and irreversible proteolytic activity mediated by executor caspases represents the ultimate outcome of both the extrinsic and the intrinsic apoptotic pathways (see below). Thus, both pathways converge on caspases-3, 6, or -7 that allow disruption of DNA and cellular components inducing the typical morphological changes in apoptosis [17]. Of note, caspases activity has been also extended to non-apoptotic functions such as cell differentiation/maturation suggesting that the caspase cascade may become activated independently of– or without inducing- an apoptotic cascade [18–20].

### Extrinsic apoptotic pathway

**Figure 1:**
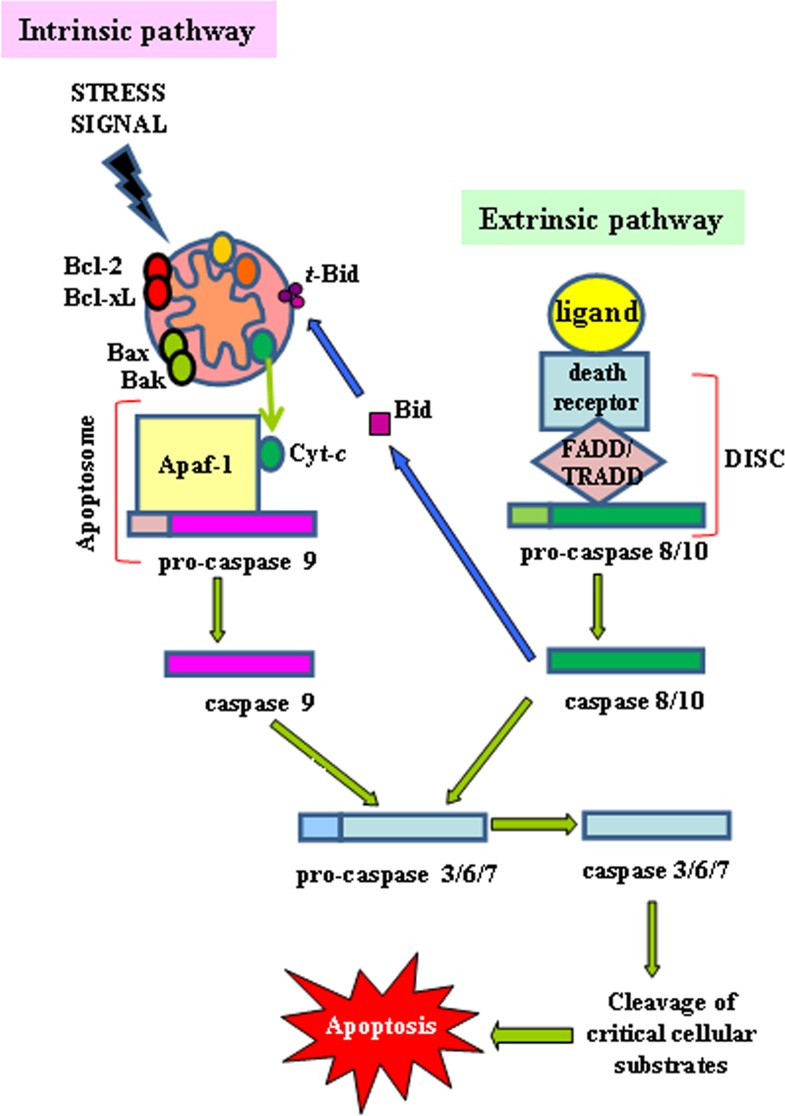
Intrinsic and extrinsic apoptotic pathways The intrinsic (mitochondrial) and the extrinsic (ligands/death receptors) cell death pathways and their convergence through t-Bid are depicted (see text for details)

The extrinsic apoptotic pathway (death receptor-dependent) is initiated by the interaction of cell surface exposed death receptors, belonging to the superfamily of tumor necrosis factor receptor (TNFR), with their respective protein TNF family ligands [21]. Death receptors are structurally defined by an intracellular protein-protein interaction domain, called the death domain (DD), which is critically involved in apoptosis-inducing signalling [22]. The more broadly characterized signaling systems of death receptor-ligands include TNFR1-TNFα, FAS (CD95, APO-1)-FasL, TRAILR1 (DR4)-TRAIL, TRAILR2 (DR5)-TRAIL. Upon death receptor stimulation by its corresponding ligand, the same receptor undergoes oligomerization and a conformational change to reveal its cytoplasmic DD to support homotypic interactions with other DD-containing proteins [21]. The role of adapter proteins (FADD/TRADD) is to sequester, at level of this protein complex, the initiator pro-caspase-8 and/or -10 resulting in the formation of the so-called death-inducing signaling complex (DISC), increasing the local concentration of pro-caspase and promoting the mutual auto-activation [23]. The activation of initiator caspases results in the processing of the downstream effector caspases-3, -6 and -7 whose activation leads to the cleavage of essential substrates for cell viability, inducing cell death (Figure 1) [17]. Some cells do not die in response to the extrinsic pathway alone and require an amplification step that is induced by caspase-8. In this situation, capase-8 targets the BH3-only protein Bid (BH3-interacting-domain death agonist) for cleavage and generate the activated fragment t-Bid; t-Bid then directly activates pro-apoptotic multi-domain proteins to induce mitochondrial outer membrane permeability (MOMP), so this co-engages the intrinsic pathway [3] (Figure 1) (see below).

### Intrinsic apoptotic pathway

The intrinsic apoptotic pathway (mitochondria-dependent) is mediated by intracellular signals that converge at the mitochondrial level in response to different stress conditions (i..e, irradiation, treatment with chemotherapeutic agents, etc.) [24]. Internal stimuli such as irreparable genetic damage, hypoxia, extremely high concentrations of cytosolic Ca^+^ and severe oxidative stress are some triggers of the initiation of the intrinsic mitochondrial pathway [25]. Subsequent activation of pro-apoptotic BH3-only members of the Bcl-2 family (Bax, Bak) neutralizes the antiapoptotic proteins Bcl-2, Bcl-xL, and Mcl-1, leading to disruption of mitochondrial membrane outer membrane permeability (MOMP) so that proteins normally confined in the intermembrane space spread into the cytosol. These proteins include the so-called apopto-genic factors, such as cytochrome-*c*, which plays a crucial role in activating the mitochondrial-dependent death in the cytosol [26]. Cytochrome-*c* binds to the cytosolic Apaf-1 (apoptosis protease activating factor-1) and triggers the formation of a complex named apoptosome, which recruits initiator pro-caspase-9 to its caspase recruitment domain (CARD), allowing auto-activation and then proteolysis. The process in turn activates downstream executor caspases-3, -6 and -7 for cleavage of cellular substrates leading to apoptotic cell death (Figure 1) [27,28].

### The B-cell lymphoma 2 (Bcl-2) family proteins

**Figure 2:**
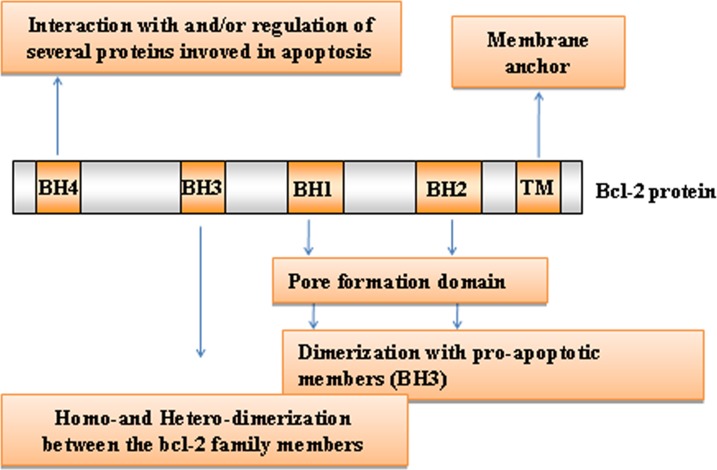
Bcl-2 family members domain composition and function Typical BH (Bcl-2 Homology) domains, listed from BH1 to BH4, are shown. TM: transmembrane domain.

**Figure 3:**
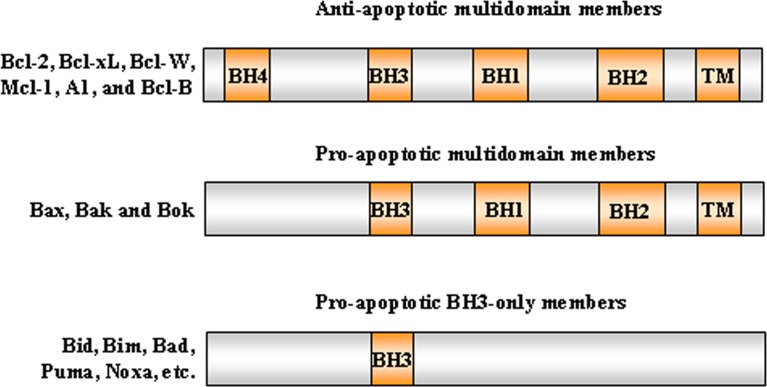
Bcl-2 protein subgroups The Bcl-2 proteins are classified into three subgroups, one group with anti-apoptotic and two with pro-apoptotic function, depending on the composition of the typical BH domains, listed from BH1 to BH4. Representative members of each subfamily are shown.

The intrinsic pathway is closely regulated by the B-cell lymphoma 2 (Bcl-2) family of intracellular proteins. This proteins family regulates both pro-apoptotic and anti-apoptotic intrinsic pathways controlling the alteration of MOMP [29]. Therefore, by mediating per-meabilization of the mitochondrial membrane, the Bcl-2 proteins serve as an “apoptotic switch” [30]. The Bcl-2 proteins are classified into three subgroups, one group with anti-apoptotic and two with pro-apoptotic function, depending on the composition of typical BH (Bcl-2 Homology) domains, listed from BH1 to BH4 [31,32] (Figure 2). Whereas the BH1 and BH2 domains of bcl-2 are required for dimerization with pro-apoptotic proteins, BH3 domain is crucially important to the interaction between pro-apoptotic and anti-apoptotic proteins and is contained by all family members. The amino-terminal BH4 domain is mainly found in the bcl-2 family members with death-repressing activity, but is also present in some pro-apoptotic molecules. The anti-apoptotic multi-domain group includes Bcl-2, Bcl-xL, Bcl-W, Mcl-1, A1, and Bcl-B, containing from three to four BH domains; the pro-apoptotic multi-domain group includes Bax, Bak and Bok proteins, containing three BH-domains (BH1, BH2 and BH3); and the pro-apoptotic BH3-only proteins group includes Bid (BH3 interacting-domain death agonist), Bim (Bcl-2-like protein 11), Bad (Bcl-2-associated death promoter), Puma (p53 upregulated modulator of apoptosis), Noxa, BMF, HRK and BIK (Figure 3) [33]. While the anti-apoptotic proteins regulate apoptosis by blocking the mitochondrial release of cytochrome-*c*, the pro-apoptotic proteins act by promoting such release.

The balance and protein-protein interactions between Bcl-2 family members is required to determine whether a cell undergoes cell survival or apoptosis. The activation of Bax (cytosolic protein that translocates into mitochondria during induction of apoptosis), and Bak (integral membrane protein located in the mitochondria and endoplasmic reticulum) involves conformational changes that trigger the formation of homo-oligomeric protein complexes that end up altering the mitochondrial membrane permeability [34,35]. The pro-apoptotic BH3-only proteins act upstream of this event binding with high affinity to anti-apoptotic Bcl-2 family members thereby allowing Bax/Bak to elicit MOMP and activation of the caspase cascade [36,37]. Anti-apoptotic multidomain members of the Bcl-2 protein family not only counteract the pore-forming activity of Bax and Bak by engaging in direct inhibitory interactions, but also prevent the generation of pro-apoptotic cytosolic Ca^2+^ waves either by reducing capacity of endoplasmic reticulum (ER) Ca^2+^ storage, an effect that is antagonized by Bax and Bak or by interacting with inositol 1,4,5-trisphosphate (IP3) receptor [38,39]. Other apoptotic factors that are released from the mitochondrial intermembrane space into the cytoplasm include apoptosis inducing factor (AIF), second mitochondria-derived activator of caspase (Smac), direct IAP Binding protein with Low pI (DIABLO) and Omi/high temperature requirement protein A (HtrA2) [40].

### The inhibitors of apoptosis proteins (IAPs)

**Figure 4:**
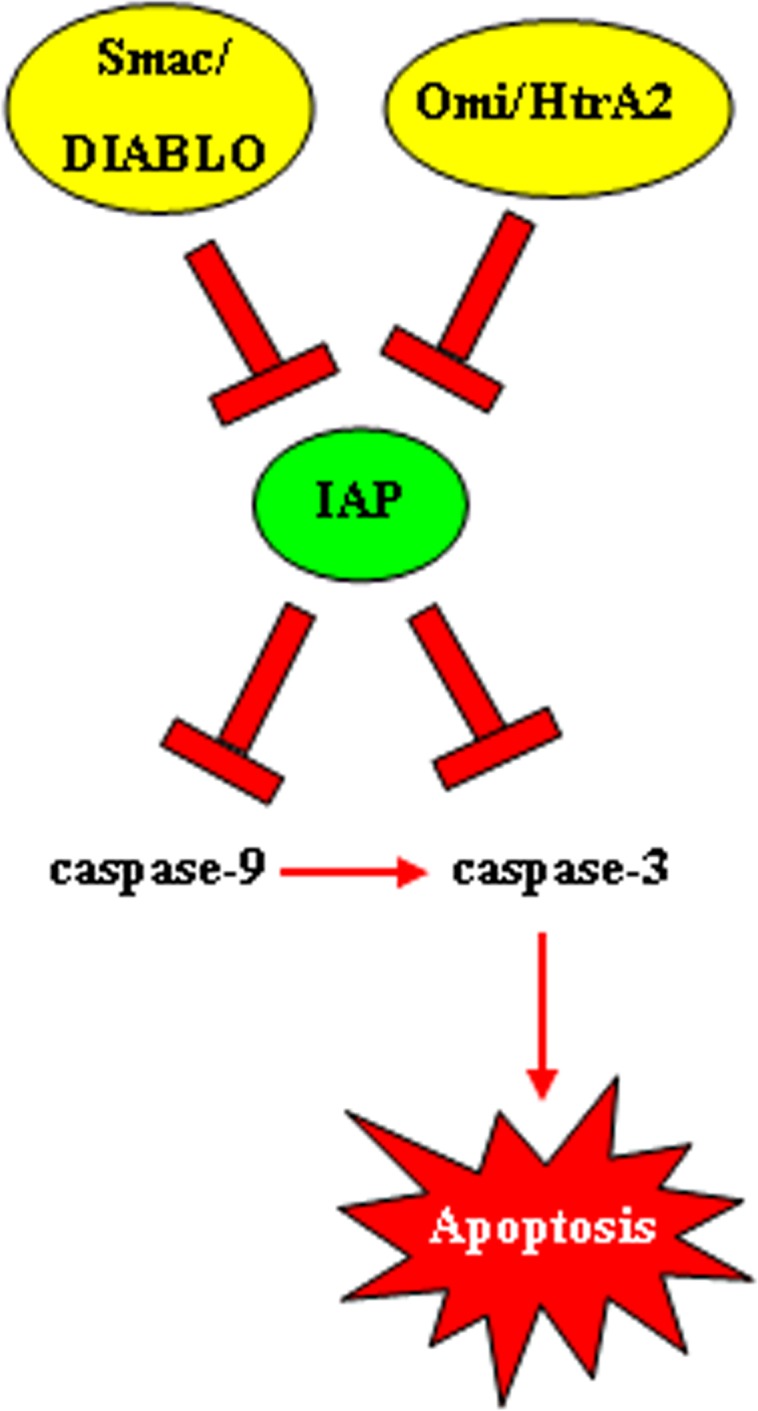
Function of inhibitors of apoptosis *proteins* (IAPs) IAPs are often overexpressed in cancer and they have the ability to bind and inactivate caspases 9 and 3. The activities of IAPs, on the other hand, may be suppressed by mitochondrial proteins, such as Omi/HtrA2 and Smac/DIABLO, released into the cytosol during apoptosis.

Considering that proteolysis is an irreversible process, strict control of caspases-mediated proteolytic cleavage is imperative to prevent inappropriate cell destruction [41]. Negative regulation of caspases function is achieved by IAP proteins family whose principal members in humans are NAIP (BIRC1), cIAP1 (BIRC2), cIAP2 (BIRC3), X-linked IAP (XIAP, BIRC4), Survivin (BIRC5), Apollon (BRUCE, BIRC6), Livin/ML-IAP (BIRC7), and IAP-like protein 2 (ILP2 – BIRC8) [42]. Their characteristic BIR (baculovirus IAP repeat) domain mediates the interaction with various proteins and gives them the ability to bind and inactivate caspases [43]. The activities of IAPs, however, may be suppressed by mitochondrial proteins, such as Omi/HtrA2 and Smac/DIABLO, released into the cytosol during apoptosis (Figure 4). These endogenous IAPs antagonists are able to bind to the BIR domain of IAPs reducing their ability to interact with caspase-3 or -9 thereby restoring their activity [44]. XIAP is the best characterized IAP so far and is generally recognized as the most potent endogenous caspase inhibitor. XIAP anti-apoptotic activity involves inhibition of active executor capsases as well as prevention of initiator caspase-9 activation [45].

### Alterations of the apoptotic pathways

**Figure 5:**
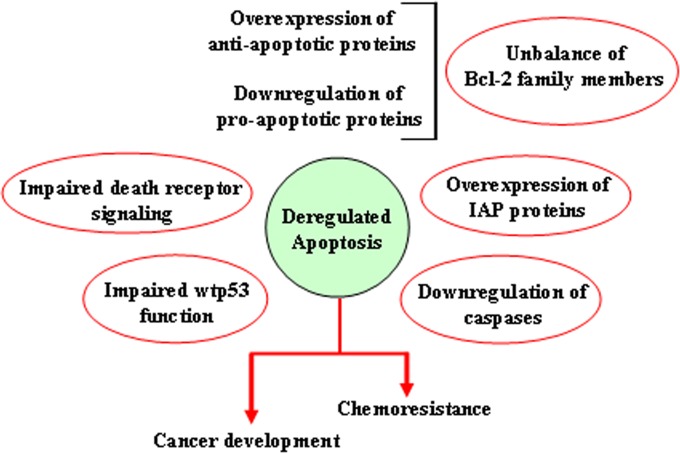
Mechanisms leading to deregulation of apoptosis Schematic representation of the different ways through which both the extrinsic and the intrinsic apoptotic pathways may be altered, resulting in reduction of apoptosis or acquisition of apoptosis resistance.

There are many ways through which both the extrinsic and the intrinsic apoptotic pathways may be altered, resulting in reduction of apoptosis or acquisition of apoptosis resistance. They include impaired death receptor signaling, disrupted balance between pro-apoptotic and anti-apoptotic proteins, reduced caspase function and impaired p53 function (Figure 5). Alteration of extrinsic apoptotic signaling has been associated with different types of human tumors, underscoring how the loss of activity of Fas-FasL system [46] or the aberrant expression of cytosolic components of this death receptor apoptotic pathway (i.e., FADD) [47] can contribute to the tumor transformation. Several genetic defects have been proven to contribute to the resistance of tumor cells to Fas-mediated apoptosis. Fas transcriptional silencing is a common oncogenic event in the epithelial transformation, while its mutation has been often associated with B-cell germinal center-derived lymphomas [48]. In acute myelogenous leukemia (AML) reduced or absent expression of FADD has been frequently observed, resulting in resistance to chemotherapy and poor patient prognosis [47,49]. Moreover, in several cancers including neuroblastoma, medulloblastoma, and small cell lung cancer (SCLC), absent or reduced expression of caspase-8 was reported [50–52]. Another resistance mechanism reported in a variety of human tumors is the overexpression of anti-apoptotic protein c-Flip, recruited at the DISC level, that prevents the pro-caspase-8 auto-activation thereby rendering cell resistant to death receptor-mediated apoptosis [53–55].

As for the extrinsic pathway, alteration of some components of the intrinsic apoptotic pathway can play a fundamental role in the development of resistance to chemotherapy in different types of tumors. Disruption in the balance of anti-apoptotic and pro-apoptotic members of the Bcl-2 family results in deregulated apoptosis in the affected cells. This can be due to overexpression of one or more anti-apoptotic proteins or downregulation of one or more pro-apoptotic proteins or a combination of both. Anti-apoptotic Bcl-2 over-expression has been reported in several human cancers, including prostate cancer, diffuse large B-cell lymphoma (DLBCL), melanoma, etc. [56–58], resulting in protection of cancer cells from apoptosis or inhibition of TRAIL-induced apoptosis [59,60]. Overexpression of Bcl-xL has also been reported in colorectal cancer and Kaposi's sarcoma [61,62]. Such overexpression confers a multi-drug resistance phenotype in tumor cells and prevents them from undergoing apoptosis [63]. Thus, high expression levels of anti-apoptotic proteins Bcl-2 and Bcl-xL have been reported to correlate with cisplatin resistance and tumor recurrence in different cancers including non-small cell lung cancer (NSCLC), head and neck, ovarian, and breast [64–68]. On the other hand, mutations in the pro-apoptotic Bax gene have been reported in colorectal cancers and contribute to resistance to anticancer treatments [69]. Increased Bcl-2/Bax ratio has been reported in chronic lymphocytic leukaemia (CLL) patients. [70]. Other examples of alteration of the intrinsic pathway include reduced expression of the basic component of the apoptosome, Apaf-1, in melanomas [71,72], as result of promoter aberrant methylation. In addition, tumor cells resistance to apoptosis also occurs as a result of alteration of mediators that control the intrinsic apoptotic pathway downstream from the apoptosome formation, i.e. acting on caspase activity. In this regard, high level of IAPs expression has been found in different types of cancers, and this evidence is considered a marker of poor prognosis for patients [73,74].

### Pharmacological targeting of the apoptotic pathways

Based on this evidence, restoration of apoptotic pathway by drugs targeting both apoptotic pathways constitutes a promising anticancer therapeutic approach. Regarding the extrinsic pathway, the down-regulation of c-Flip by metabolic inhibitors and the promotion of caspase-8 activation by interferon, are some examples of strategies aimed at making tumors responsive to death receptor-induced apoptosis, and more generally, to chemotherapy-induced apoptosis [55,75,76]. The therapeutic importance of inducing apoptosis through the extrinsic pathway also extends to cancer cells that do not show defects in components of that pathway. Indeed, inducing the apoptosis by stimulating the extrinsic pathway can overcome the resistance to therapeutic agents that act by causing DNA damage, as death receptor-dependent apoptosis may occur regardless of the stress response. An example of such therapeutic strategy is represented by the ligand TRAIL known to induce apoptosis in different tumor cell lines [77]. The preferential destructive effect against tumor cells and the apparent absence of systemic toxicity through TRAIL-induced apoptosis, led to the development of antibodies with agonistic activity against the TRAIL death receptors (DR4 and DR5) or soluble recombinant derivatives of TRAIL (sTRAIL) as promising chemotherapeutic agents [78]. An attractive strategy to sensitize resistant malignancies to TRAIL-induced cell death is the design of small molecules that target and promote caspase-8 activation. Through an *in silico* screening some authors successfully found a small molecule activator of caspase-8 [79]. Experimental validation performed in multiple cell lines, such as leukemic and prostate cells, revealed that CaspPro small molecule promotes caspase-8 activation, caspase-3 activation and PARP cleavage, in the presence of TRAIL, leading to cell death [79]. Owing to its different toxicity for transformed as opposed to normal cells, Apo2/TRAIL shows promise as potential cancer therapy agent [80,81].

As in the extrinsic pathway, mediators of the intrinsic pathway involved both in tumorigenesis and chemo-resistance, are targeted for therapeutic approaches. These anticancer strategies attempt to develop drug-designed inhibitors of anti-apoptotic proteins typically overexpressed in cancer cells, such as Bcl-2, Bcl-xL and IAPs [82]. Efforts to target Bcl-2 proteins involve the development of agents that disrupt Bcl-2 complexes. BH3 mimetics bind to the hydrophobic groove of antiapoptotic proteins mimicking the action of BH3-only proteins in binding to pro-survival proteins, leading to the release of BH3-only proteins from complexes and activation of BAX and BAK. So far, nearly a dozen BH3 mimetics are under investigation as Bcl-2 inhibitors in different phases of human clinical trials such as AT-101 (R-(−)-gossypol) [83,84], ABT-199 (venetoclax) [85], ABT-737 [86], ABT-263 (navitoclax, orally available derivative of ABT-737) [87,88], GX15-070 (obatoclax) [89,90] and TW37 [91]. The field of inhibitors of Bcl-2 family members is in continuous development [92,93], underscoring the importance of these molecules as potent anticancer agents. Moreover, targeting the specific BH4 domain of Bcl-2 is also emerging as a novel strategy for anticancer therapy [94]. Thus, Bcl-2, via its BH4 domain, cooperates with numerous proteins regulating different cellular pathways involved in tumor progression and chemoresistance such as hypoxia and angiogenesis [95–97]. Recently, a small molecule namely BDA-366 was discovered as a potent and effective BH4 domain antagonist, displaying remarkable anticancer activity in vitro and in vivo, thus providing the proof-of-concept of this approach [98]. Another innovative approach to inhibit Bcl-2 comes from the evidence that human bcl-2 gene contains a GC-rich sequence located in its promoter with the potential to form G-quadruplex structures [99] and functions as a transcriptional repressor element. Therefore, G-quadruplex-specific ligands can regulate the transcription of bcl-2 through stabilization of quadruplex structure [100,101].

Interestingly, it has been shown that the tumor suppressor p53, at least in part by transcription independent mechanisms, contributes to cell death induction by BH3 mimetic inhibitors of BCL-xL. In addition to mildly facilitating the ability of compounds to derepress BAX from BCL-xL, p53 also provides a death signal downstream of anti-apoptotic proteins inhibition that is independent from PUMA, as enhanced p53 can substitute for PUMA to promote BAX activation in response to BH3 mimetics [102]. It is thus of particular relevance that p53, even when expressed constitutively under conditions where it does not influence the expression of its pro-apoptotic transcription targets, enhances cell death induced by BCL-xL inhibition [102]. Such results suggest on one hand that BH3 mimetics may not totally substitute for the lack of an active p53 tumor suppressor in cancer cells; on the other hand, they imply that healthy tissues may be more harmed than anticipated when BCL-xL inhibitors are combined with chemotherapeutic agents that even mildly affect p53.

Among the therapeutic strategies targeting IAPs two approaches have being developed, that is the use of antisense oligonucleotides and of small-molecule inhibitors. The XIAP down-regulation through administration of antisense agents carried by an adenoviral vector has been proven effective in inducing apoptosis in chemoresistant ovarian cancer cells [103] and sensitizing lung cancer cells to the radiation treatment [104]. Similarly, the inhibition of XIAP expression with specific oligomers has been shown to induce caspase-3 activity, boosting the apoptotic effect of cisplatin and TRAIL in human prostate androgen-insensitive cancer cells [105]. Moreover, preclinical studies have shown that Smac mimetics can directly trigger cancer cell death or sensitize tumor cells to various cytotoxic therapies, including conventional chemotherapy, radiotherapy, or novel agents. They promote activation of caspases by neutralizing XIAP-mediated caspase inhibition [106]. Therefore, the success of each therapeutic strategy depends mainly on the ability of the therapeutic tool to induce apoptosis either by targeting the overexpressed anti-apoptotic proteins or by stimulating the expression of the pro-apoptotic molecules.

However, it is worth to mention that the cancer genetic background may induce failure of apoptosis by drugs. In this regard, KRAS and the PI3K/AKT/mTOR pathway are frequently dysregulated in cancer and, for such reason, are the most critical targets in clinical oncology. Thus, direct targeting of KRAS has not been successful so far and, similarly, inhibition of the PI3K/AKT/mTOR pathway often results in apoptosis resistance. Using a panel of 20 human KRAS-mutant NSCLC (non-small cell lung cancer) cell lines Hata and collaborators show that most human KRAS-mutant cell lines fail to undergo marked apoptosis in response to AZD6244 (Selumetinib, a potent, selective, and ATP-uncompetitive inhibitor of MEK1/2 kinases) [107] in combination with GDC-0941 (an orally bioavailable inhibitor of class I PI3K) [108], thus suggesting that failure to induce apoptosis may limit the efficacy of combined MEK and PI3K inhibition for KRAS-mutant NSCLCs. This differential apoptotic response induced by MEKi/PI3Ki is not simply explained by variable inhibition of RAS effector pathways but results from differential ability of the MEK and PI3K pathways to modulate the BCL-2 family of apoptotic regulatory proteins [109]. Another recent study reveals that Bcl-xL upregulation is an important mechanism of apoptosis resistance in mutant KRAS cells. Concurrent induction of pro-apoptotic Noxa/Bik and antagonism of Bcl-xL have been shown to synergistically interact to overcome KRAS-mediated apoptosis resistance [110]. These findings highlight a promising therapeutic strategy to overcome apoptosis resistance in KRAS-mutant colorectal cancer cells. Moreover, Corcoran and collaborators identified, by a pooled shRNA-drug screen, a synthetic, lethal interaction of combined Bcl-xL and MEK inhibition to promote tumor regressions in KRAS mutant cancer models [111]. Therefore, a dual-targeted or multitargeted strategy may be more efficient to overcome the resistance due to cancer genetic background.

### Oncosuppressor p53 and apoptosis

**Figure 6:**
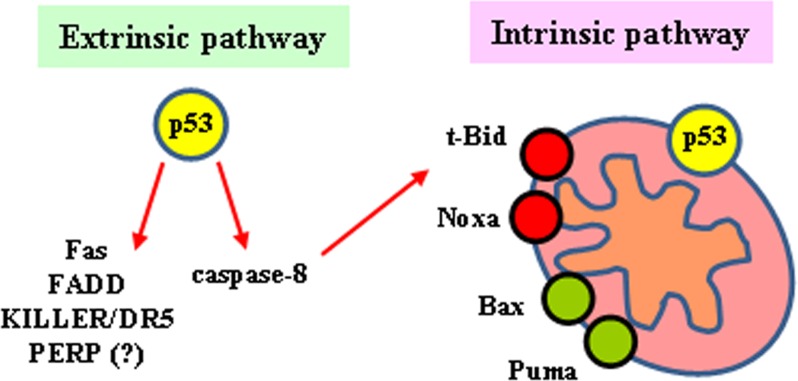
p53-mediated apoptosis Role of p53 in both the extrinsic and the intrinsic pathway and their convergence through t-Bid.

The tumor suppressor p53 is a transcription factor that, upon DNA damage, is activated to induce sequence-specific target genes involved in either cancer cell growth arrest or apoptosis [112]. Activation of wild-type (wt) p53 occurs in response to genotoxic stress essentially through posttranslational modifications, such as acetylation and phosphorylation, resulting in protein stabilization (by escape from proteasome-mediated degradation) and nuclear localization leading to binding to sequence-specific promoters of target genes as final outcome of its function as transcription factor [113]. The induction of apoptosis by p53 in response to cellular stress is its most conserved function and is crucial for p53 tumor suppression [114]. The apoptotic activation of p53 is central not only for preventing tumor transformation but also for efficient response to therapies aiming at tumor eradication. In response to cellular stress p53 regulates molecules involved in both the death receptor (extrinsic) and mitochondria-dependent (intrinsic) apoptotic pathways [115]. In response to multiple chemotherapeutic drugs two pro-apoptotic members of the TNFR superfamily, Fas/Apo1 and Killer/DR5, are regulated in a p53-dependent manner [116,117]. In addition to stimulating Fas transcription, activated p53 may enhance levels of Fas at the cell surface promoting trafficking of the Fas receptor from the Golgi [118]. Another membrane-bound protein that was identified as a p53 target gene is p53 apoptosis effector related PMP-22 (PERP), although the precise mechanism by which its induction occurs has not being fully elucidated [119] (Figure 6). Regarding the apoptotic function of the intrinsic pathway, p53 seems to play a pivotal role because it modulates both pro-survival and pro-apoptotic Bcl-2 family members. Indeed, a key subset of the Bcl-2 family genes are p53 targets, including *Bax*, *Noxa*, *PUMA* and *Bid* [120–122] (Figure 6). *PUMA* gene is extremely effective in inducing apoptotic cell death within few hours and, more importantly, knockout experiments in human colorectal cancer cells showed that *PUMA* is required for p53-induced apoptosis [123]. Moreover, p53 appears to promote the convergence of the intrinsic and extrinsic pathways through *Bid* regulation. Indeed, *Bid* gene has been found to be transcriptionally induced by p53 in response to γ-irradiation [124]. Interestingly, cellular chemo-sensitivity to the DNA-damaging agents doxorubicin and 5-fluorouracil appears to be critically dependent on the presence of wtp53 and Bid. Therefore, the induction of Bid by p53 helps to sensitize the cells to the toxic effects of chemotherapeutic drugs [124]. While the induction of some p53 target genes appears to be sufficient to initiate apoptosis, another class of p53 target genes (i.e., Apaf-1, caspase-6, and Bid) does not efficiently induce apoptosis per se but rather sensitizes cancer cells to the effects of chemotherapeutic agents, improving the apoptotic outcome [124–127]. Moreover, p53 also participates in apoptosis induction in a transcription-independent way by acting directly at mitochondria [128]; mechanistically, p53 interacts with anti-apoptotic Bcl-xL as well as pro-apoptotic Bcl-2 family proteins resulting in releasing of the pro-apoptotic effectors Bax/Bak that elicit cytochrome-*c* release and procaspase-3 activation [129].

### Waking up the guardian: p53 as a druggable target

Because of its critical antitumor function, p53 is frequently targeted for inactivation and suffers disabling mutations or deletions in about 50% of all malignant tumors. The other half of human cancers express wild-type p53 protein that, however, can be inactivated by deregulation of regulatory proteins [130]. Stimulation of disabled p53 pathways has been suggested as a valuable anticancer strategy and, interestingly, activated wtp53 may target cancer cells though sparing the normal ones [131] which is an important concern in clinical studies. The p53 oncosuppressor protein is normally kept at low level because subject to negative regulation by MDM2-dependent proteasome degradation [132]; in response to genotoxic stress, however, p53 undergoes post-translational modifications that allow the protein to escape MDM2 control, accumulate, and become active [133]. The *mdm2* gene is amplified in a significant proportion of human tumor types, thereby contributing to tumor development by efficiently reducing the availability of a functional p53 protein [134]. The MDM2-negative regulation of p53 protein can be neutralized by specific protein modifications such as serine 46 (Ser46) phosphorylation [135], a key determinant in shifting the p53 pro-apoptotic transcription in response, for instance, to UV irradiation and chemotherapy [136,137]. In particular, p53-Ser46 phosphorylation by kinase HIPK2 is able to neutralize MDM2-mediated p53 inhibition rescuing p53 transcriptional activity of pro-apoptotic factors such as *p53AIP1*, *PIG3*, *Bax*, *Noxa*, *Puma* and *Killer/DR5* [138–142]. The interaction between p53 and MDM2 is a promising target in anticancer therapy [143]. To this aim, various peptidomimetic small molecules have been developed as protein-protein interaction blockers [144]. Among these is Nutlin-3, an imidazoline-based MDM2 antagonist that potentially inhibits the p53/MDM2 interaction though maintaining MDM2 E3 ligase activity [145]. The pharmacological action of Nutlin-3 is through both the transcription-dependent and -independent p53 apoptotic pathways [128,146,147]. MDM2 can also trigger, in response to low genotoxic damage, the downregulation of p53 apoptotic activator HIPK2 [148]. In agreement, the use of Nutlin-3 has been shown to mainly induce mitotic arrest rather than apoptosis [149]. Interestingly, co-treatment of cancer cells with zinc ion in the presence of Nutlin-3 can interfere with the interplay between HIPK2, p53 and MDM2 favoring HIPK2 stabilization and induction of p53 apoptotic activity through inhibition of MDM2 ligase activity [150]. In addition, p53 apoptotic activation can be achieved by zinc combination with low-dose doxorubicin (ADR) that used alone does not achieve such effect; mechanistically, zinc supplementation reduces the p53 binding to MDM2, improving the low-dose drug-induced cytotoxic effect and cancer cell apoptosis [151]. Additionally, zinc ion restores the HIPK2/p53 apoptotic pathway that is inhibited by hypoxia [152]. Co-treatment with Nutlin-3 and Bcl-2 inhibitor ABT-737 has been shown to greatly enhance the sensitivity to apoptosis of cancer cells with high MDM2 levels [153], suggesting that the combined inhibition of MDM2 and Bcl-2 could be a multi-target-based anticancer strategy to trigger tumor death [154]. Some p53 activators as small-molecules MDM2 antagonist are in clinical trials [155] ( https://clinicaltrials.gov). In contrast with the majority of the approaches that target the interaction between p53 and MDM2, a new method has been developed aimed at inhibiting the activity of the MDM2/MDM4 complexes by interfering with their heterodimerization [156]. The binding of the peptide mimicking the MDM4 C-terminus tail to MDM2 impairs MDM2-mediated p53 ubiquitination and activates p53-dependent transcription and oncosuppressive activities [156]. MDM4 (also known as HDM4, MDMX or HDMX) is a cytoplasmic protein with p53-activating function under DNA damage conditions. Particularly, MDM4 promotes mitochondrial localization of p53 phosphorylated at Ser46 through MDM4/HIPK2/p53 cytoplasmic assembly, uncovering coordinated repression of molecules with anti-apoptotic activity such as Bcl-2, release of cytochrome-*c* and apoptosis [157,158]. The existence of nuclear and cytoplasmic complexes able to stimulate the same p53 modification, that is Ser46^P^, may indicate the presence of overlapping pathways to ensure the proper realization of a crucial process as the apoptosis. These findings highlight the potential therapeutic value of targeting the MDM2/MDM4 heterodimers for p53 apoptotic function.

Pharmacological reactivation of mutant (mut) p53 is an interesting field of research under continuous development aimed at designing new drugs. Some of them exploit the intrinsically unstable nature of mutp53 and therefore the possibility to stabilize the wild-type conformation thus restoring wild-type function and tumor response to therapies. Numerous findings about this subject have been shown and summarized in different reviews [159–161].

### MicroRNA and apoptosis

MicroRNAs (miRNAs) are highly conserved, small noncoding RNA molecules, which post-transcriptionally regulate gene expression via inhibition of mRNA translation or inducing degradation of target mRNAs [162]. They are key regulators of many cell processes often deregulated in cancer, including apoptosis. Indeed, it is becoming clear that miRNAs might act as both anti-apoptotic and pro-apoptotic by directly targeting, respectively, pro- or anti-apoptotic mRNAs or their positive regulators [163]. The currently known apoptosis-regulating miRNAs list is expected to expand quickly and hopefully also their therapeutic use; therefore, we just highlight here some miRNAs involved in apoptosis regulation. Among the microRNAs involved in regulating the extrinsic apoptotic pathway, miR-20a, miR-21, miR-196b and miR-590 were reported as potential modulator of Fas/FasL system in different cancer types [164–167], while MiR-34a, miR-181c and miR-187 were shown to directly target TNF-α mRNA [168–170]. Among the microRNAs involved in regulating the intrinsic pathway there are the let-7 family, miR-15a, miR-16-1, miR-204, and miR-608, just to mention a few. The Let-7 family is highly conserved in sequence across animal species and is one of the first identified miRNA families. Let-7 miRNAs have been shown to negatively regulate Bcl-xL expression in human hepatocellular carcinomas and induce apoptosis in cooperation with anti-cancer drug targeting Mcl-1 [171]. Bcl-2 mRNA may be targeted by miR-204 with consequent increase in cells responsiveness to both 5-fluorouracil and oxaliplatin treatments and therefore to apoptotic cell death [172]. MiR-608 has been reported to target Bcl-xL in chordoma malignancy and lung cancer [173]. Notably, numerous miRNAs are also transcriptionally modulated by wtp53 [174] and among them is miR-34a [175,176], a member of the MiR-34 family implicated in cell death/survival signaling. MiR-34a is associated with G1 cell cycle arrest, senescence and apoptosis, thereby possessing a tumor suppressor activity. Deregulation of MiR-34a has been reported in several types of cancers [175,176]. Mutant (mut) p53 was also found to play a role in the regulation of miRNA processing. Garibaldi and collaborators demonstrate that mutp53 proteins modulate the biogenesis of several miRNAs in cancer cells directly interfering with Drosha-p72 association and promoting cell survival and cell migration [177]. They demonstrate a global impact of mutp53 on miRNA biogenesis and suggest that miRNAs are downregulated by mutp53 in order to inactivate tumor suppressive pathways. Of note they found that the endogenous wtp53 has an opposite effect on the expression of mutp53 repressed miRNAs on colon cancer cell lines confirming the contribution of mutp53 gain of oncogenic function (GOF) on miRNA repression [177]. Additional studies on a large scale would help in identifying the entire repertoire of miRNAs negatively downregulated by different mutp53 in different tumor models. According to the authors, the characterization of the entire gene-regulatory networks governed by mutp53-miRNA cross-talks will offer a molecular basis for diagnostic and therapeutic strategies based on miRNA biology. In the meanwhile, developing strategies to block mutp53 GOF may have clinical impact in cancer treatment.

Delivery of miRNAs as synthetic miRNA mimics or antagomirs has emerged as a promising approach to treat cancer. Although different miRNAs are currently in the preclinical stage and ready to enter Phase 1 clinical trials, to date, only two miRNA therapeutics are registered for the treatment of cancers [https://clinicaltrials.gov]. The first therapeutic trial began in 2013 and is a Phase I, open-label, multicenter, dose-escalation study to investigate the safety, pharmacokinetics and pharmacodynamics of MRX34 in patients with unresectable primary liver cancer or advanced/metastatic cancer with or without liver involvement or in patients with hematologic malignancies (Mirna Therapeutics). MRX34 is based on the formulation of miR-34 mimic and the liposomal delivery technology SMARTICLES (Marina Biotech). The second one, began in early 2015, and is an early stage clinical trial of a new therapeutical approach for selected patients with malignant pleural mesothelioma or non-small cell lung cancer. The trial aims to test optimal dose of TargomiRs, an experimental medication consisting of three components, that is, miR-16-based microRNA mimic, a nanoparticle drug delivery system using nonliving bacterial minicells, and an anti-epidermal growth factor receptor antibody as a targeting moiety. The trial is being carried out in three different hospitals in Australia and the study is expected to be completed in mid 2016.

### Concluding remarks

Intensive investigation in the last decades on the molecular mechanisms of apoptosis in cancer cells has led to the identification of the several molecules involved in both the intrinsic and the extrinsic apoptotic pathways. This is underscored by the extensive literature that those studies have produced in the last years. Those findings also reported how the many different alterations of key players of the apoptotic mechanisms are responsible of evasion from apoptosis and therefore of tumor development and resistance to therapies. For that reason, evasion from apoptosis is an hallmark of cancer and apoptotic proteins are interesting therapeutic targets. Therefore, this insight into the deregulation of apoptosis has focused the research attention towards the development of apoptosis-reactivating strategies, to be used in the treatment of various types of cancer, that hold great promise for the benefit of patients, although the mechanisms defining their mode of action still need to be unveiled, as recently highlighted [178]. Some molecules or therapeutic strategies are in preclinical trial, others are already in clinical trials, though underscoring the usefulness of such discoveries.

However, the study of apoptosis still presents challenges that should be addressed in future studies. They include, for instance, the study of 3-D cellular models, since most of the findings have been so far produced in 2-D cell culture systems. Knowing that the tumor is a three-dimensional entity and that the environment plays a big role in the cross-talk with cancer cells, it is likely that more physiological studying approach for the manipulation of the apoptotic machinery might give us novel insight into the mechanisms of tumor development and response to therapies. Moreover, additional studies on the development of drugs aiming at targeting, for instance, IAP proteins or mutp53 should take in consideration also the in vivo toxicity and the fact that they should selectively induce apoptosis in malignant and not in normal cells. In conclusion, there is little doubt that drugs that target the deregulated apoptotic pathways could have wide application in the treatment of cancer. The intense effort devoted lately to target the apoptotic pathway is encouraging and supportive for the development of new approaches to drug discovery and therapy.
